# Severe Hemolytic Crisis in Uncontrolled AIHA Triggered by Dengue

**DOI:** 10.1155/carm/8165658

**Published:** 2025-03-29

**Authors:** Christian Alberto Rodriguez-Saldaña, Sofía Cavalcanti-Ramírez, Carmen Claudia Quesada-Osoria, Luis Gabriel Farfan-Chavez, Guiovanna María Wong-Terrones, Helena Karin Dominguez-Troncos

**Affiliations:** ^1^José Cayetano Heredia Hospital III-1 EsSalud, Piura, Peru; ^2^Department of Infectious Diseases, José Cayetano Heredia Hospital, Castilla, Piura, Peru; ^3^Department of Pulmonology, José Cayetano Heredia Hospital, Castilla, Piura, Peru; ^4^Department of Internal Medicine, José Cayetano Heredia Hospital, Castilla, Piura, Peru

**Keywords:** autoimmune anemia, dengue, hemolytic anemia

## Abstract

We present the case of a woman with a history of autoimmune hemolytic anemia (AIHA), untreated for 1 year, who presented with fever, myalgia, arthralgia, anemia, and thrombocytopenia, along with a positive nonstructural protein antigen 1 (NS1Ag) test. During the course of dengue, the patient developed severe hemolytic anemia, posing a potentially life-threatening scenario due to the rapid decline in hemoglobin levels and the risk of multiorgan failure. Treatment with methylprednisolone was initiated alongside supportive care for dengue, and balancing corticosteroid risks in a viral infection was crucial to stabilize the patient, ultimately leading to a favorable outcome. This case highlights the importance of prompt diagnosis and integrated management of autoimmune diseases and viral coinfections in dengue-endemic areas.

## 1. Introduction

Dengue is endemic in tropical regions such as Oceania, Southeast Asia, and South America, and it is projected to impact over 200 countries in the near future [1]. The 2023 dengue outbreak in Peru was the largest recorded, with 222,620 cases between January and July, 10 times the annual average of the previous five years. There were 381 deaths, with a mortality rate of 0.17%, and more than half of the fatalities were over 60 years old. The most affected regions were Piura, Lambayeque, and La Libertad. The most common serotypes were DENV-2 and DENV-1, both with a 49% incidence [2].

In AIHA, the immune system produces antibodies that destroy red blood cells, primarily in the spleen, causing hemolysis and anemia [3]. Symptoms include fatigue, weakness, and jaundice. The pathogenesis is complex, involving *T-* and *B*-cell dysfunction, an imbalance between Th17 cells and Tregs, and increased oxidative stress, contributing to premature erythrocyte destruction [4]. Hemolysis can be intravascular or extravascular, depending on the type of antibody involved, and is associated with various underlying conditions, such as autoimmune diseases and infections [5]. Dengue can precipitate or exacerbate AIHA, leading to a rapid decline in hemoglobin levels that, if untreated, may result in severe complications such as multiorgan failure or hemorrhagic shock. This occurs through mechanisms such as antibody-dependent enhancement (ADE), inflammasome activation by cytokines such as IL-18, and cross-reactivity with self-antigens, which trigger cytokine storms and amplify hemolysis [6–8]. Reports have documented autoimmune reactivation during dengue infections in conditions such as Guillain–Barré syndrome and systemic lupus erythematosus, further emphasizing the virus's role in triggering crises in preexisting autoimmune diseases [9]. While dengue infection in patients with AIHA is not uncommon, few cases in the literature describe severe hemolytic crises from untreated AIHA for 1 year combined with dengue.

In light of Peru's largest dengue epidemic, multiple cases of hematological, rheumatological, and oncological diseases were observed to debut or reactivate during or after dengue infection.

## 2. Clinical Case

A 75-year old female patient, a housewife by occupation, from Piura with a history of autoimmune hemolytic anemia (AIHA) diagnosed 10 years ago, under irregular treatment with prednisone 10 mg, folic acid 1 mg, and pyridoxine 50 mg orally every 24 h (no hematology clinic follow-up for 1 year). The patient reported a prolonged period without hemolytic crises or symptoms during this time. She also has a 10-year history of hypertension, treated with Losartan 50 mg orally every 12 h, and osteoarthritis, treated with methotrexate 7.5 mg daily for two days a week and leflunomide 20 mg orally daily. She denies a history of drug allergies.

On November 16, 2023, at 19:08, she was admitted to the emergency department with a 3-day history of malaise, weakness in the lower limbs, drowsiness, and abdominal discomfort of sudden onset and progressive course. Initial evaluation showed blood pressure (BP) of 117/75 mmHg, heart rate (HR) of 86 bpm, and oxygen saturation (SpO2) of 98%. Warm skin and hydrated mucous membranes were observed. On abdominal palpation, a soft, depressible abdomen with pain on superficial palpation in the epigastric region was noted. Laboratory tests revealed severe anemia with hemoglobin (Hb) of 6 g/dL and thrombocytopenia of 101,000 mm^3^ (Table 1). These findings indicate anemia due to hemolysis and thrombocytopenia consistent with the critical phase of dengue. Urinalysis showed leukocytosis and microhematuria (without dysuria). Although reticulocyte evaluation was not performed during hospitalization, urinalysis findings and laboratory data, including a drop in hemoglobin (6 g/dL to 5.4 g/dL), elevated indirect bilirubin (1.46 mg/dL), and a positive direct Coombs test (+++) suggest predominantly extravascular hemolysis, with a possible minor intravascular component. The elevated bilirubin levels also support active red cell destruction during this phase. Although no clinical signs or hemodynamic changes suggested bleeding in other compartments, close monitoring is required due to the severity of dengue and thrombocytopenia. Serological tests confirmed dengue infection, with positive results for reactive NS1 and IgM.

On November 17 (Day 4), she was hospitalized in the pneumoinfectology ward with a diagnosis of dengue with warning signs (intense and persistent abdominal pain) plus associated comorbidities due to the risk of hemolysis (Group B) in the critical phase. Treatment included fluid therapy according to the WHO algorithm (dynamic according to monitoring), pyridoxine 50 mg orally daily, folic acid 15 mg orally every 12 h, hydroxocobalamin 1 mg intramuscularly every 24 h for 7 days, and losartan 50 mg orally every 12 h. Despite improvement in warning signs, general malaise and joint pain persisted.

On November 18 (Day 5), she was evaluated by hematology, which revealed severe anemia (Hb 5.4 g/dL) with normal corpuscular parameters (MCV 92.7 fL, MCH 32.7 pg, MCHC 35.3 g/dL). A direct Coombs test confirmed the diagnosis of hemolysis. Abdominal ultrasound showed chronic diffuse liver disease and mild splenomegaly. This hemolytic crisis can be classified as extravascular hemolysis, based on the positive Coombs test, stable corpuscular parameters, and elevated indirect bilirubin levels, which primarily affect mature red cells without altering immature cell populations.

On November 20 (Day 7), severe anemia persisted with Hb 5.7 g/dL and thrombocytopenia at 58,000 mm^3^. Treatment with 1 g of methylprednisolone diluted in 300 mL of sodium chloride daily for 3 days was initiated. The patient showed favorable improvement in laboratory parameters. Despite the hemolysis, MCV and MCH remained normal due to its extravascular nature, which affects mature erythrocytes without altering immature cell proportions. Blood transfusion was not administered due to the positive Coombs test, indicating active hemolysis, as transfusion could exacerbate the hemolysis. The persistent thrombocytopenia also reflects the ongoing critical phase of dengue, compounded by hemolysis. The lack of reticulocytosis may reflect a delayed bone marrow response or the impact of inflammation during corticosteroid treatment.

By November 23 (Day 10), platelet counts increased to over 100,000 mm^3^, and the patient was considered in the recovery phase. She was given an epidemiological discharge for dengue, and a reevaluation by hematology recommended administering a new pulse of methylprednisolone therapy.

**Figure 1:**
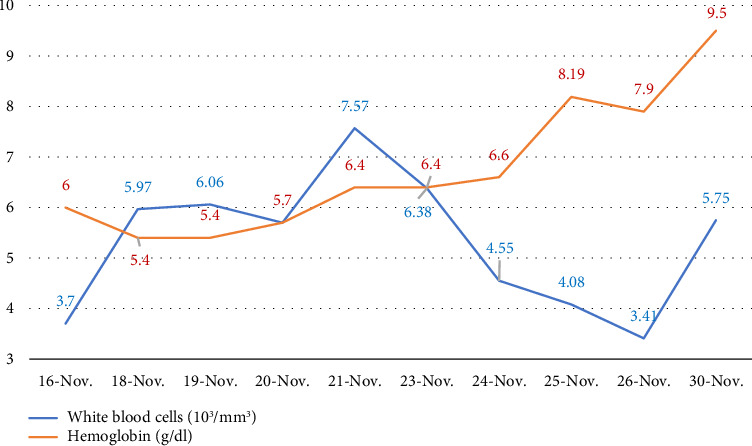
Trend line of white blood cells and hemoglobin during hospitalization.

Finally, on November 27, with hemoglobin at 7.9 g/dL, the patient was discharged. Following discharge, hematology evaluated reticulocyte levels, reporting a value of 5.8% (normal range: 0–5), after completing the methylprednisolone pulse and initiating oral prednisone (40 mg every 12 h for 5 days). Corticosteroids were gradually tapered during outpatient follow-up to a maintenance dose of 5 mg. This recovery phase was marked by improved hemoglobin levels and normalized platelet counts, indicating resolution of both hemolysis and dengue-associated thrombocytopenia. The evolution of white blood cells and hemoglobin during the hospitalization period is shown in Figure 1.

## 3. Discussion

Dengue is recognized as a viral disease capable of affecting cellular immunity, predisposing patients to superimposed bacterial infections, and activating different autoimmune processes, such as encephalomyelitis, Guillain–Barré syndrome, and triggering or exacerbating preexisting autoimmune conditions such as AIHA [10, 11]. In patients with AIHA, the onset of fever and other nonspecific symptoms in an endemic area might initially point toward a differential diagnosis, including dengue virus infection. However, in patients who have suspended regular treatment or follow-up, it is crucial to consider the possibility of an acute hemolytic crisis reactivation that could complicate the clinical picture [11]. In such cases, proactive follow-up and adherence to maintenance therapy are essential to mitigate the risk of severe complications during dengue outbreaks, particularly in tropical regions.

In this context, serological tests for dengue, along with laboratory tests for active hemolysis (elevated LDH, decreased haptoglobin, and positive direct Coombs test), are sufficient to differentiate and confirm the diagnosis, avoiding mistakes that could worsen the patient's condition [8]. In the case presented, the elevated indirect bilirubin and positive Coombs test guided the decision to refrain from blood transfusions, as this could exacerbate hemolysis due to the ongoing immune response. Instead, methylprednisolone was prioritized to control hemolysis and stabilize the patient. Severe anemia is a significant determinant in the course of dengue. In a controlled AIHA case, dengue may induce additional hemolysis, but in those with active or uncontrolled hemolysis, the risk of severe anemia is even greater. Exacerbated hemolysis can lead to a sharp drop in hemoglobin levels, compromising tissue oxygenation and increasing the risk of severe complications, such as hemorrhagic shock or multiple organ failure, thereby worsening the prognosis, as described in some reports [12, 13].

The evidence suggesting that dengue can trigger a hemolytic crisis in AIHA patients is supported by the fact that this viral infection can induce a dysregulated immune response, increasing antibody production against erythrocytes and exacerbating hemolysis in AIHA patients [14, 15]. This is particularly relevant in poorly controlled AIHA patients, as dengue may reactivate the disease, precipitating a hemolytic crisis that may present with an abrupt drop in hemoglobin levels, jaundice, and increased indirect bilirubin, complicating clinical management [8]. Reports of similar cases highlight the challenges of managing poorly controlled autoimmune diseases during dengue. In the differential diagnosis, it is crucial to exclude other potential causes of hemolysis, such as drug-induced hemolytic anemia, paroxysmal nocturnal hemoglobinuria, or infections such as malaria, which can present with overlapping symptoms. In this case, the serological confirmation of dengue alongside hemolysis-specific findings was instrumental in narrowing down the diagnosis. This case emphasizes the need for individualized strategies, particularly in elderly patients with comorbidities.

The simultaneous treatment of AIHA and dengue in elderly patients is complex and requires an integrated approach. In controlled AIHA cases, dengue treatment focuses on fluid management, considering comorbidities and symptomatic support. However, in the presence of active hemolysis, especially in poorly controlled AIHA cases, the cautious use of corticosteroids, such as methylprednisolone, is necessary, as this medication may compromise the immune response to dengue, altering the disease course and potentially prolonging the critical phase. The decision to administer corticosteroids underscores the importance of balancing the risks of exacerbated viral replication against the need to mitigate life-threatening hemolysis. Close monitoring of the patient's clinical and laboratory parameters, including hemoglobin levels, platelet counts, and signs of fluid overload, is essential for a favorable outcome. Methylprednisolone was chosen as the main treatment to manage the severe hemolytic crisis based on available evidence and resources. Alternative corticosteroid regimens were not explored, as methylprednisolone is a well-established option for this condition. Future comparisons with other regimens could further strengthen management approaches in similar cases.

Another critical aspect is fluid balance, as severe anemia can complicate fluid administration and increase the risk of pulmonary edema. Intravenous fluid administration should be closely monitored, with strict oversight of fluid balance and hourly diuresis. The dynamic monitoring of this patient, including regular adjustments based on hemoglobin trends, platelet counts, and fluid status, highlights the importance of tailored management to optimize outcomes in elderly patients with comorbidities.

## 4. Conclusions

Dengue can trigger a hemolytic crisis in AIHA patients, aggravating anemia and complicating clinical management. In tropical regions like ours, where dengue is endemic, during outbreaks or epidemic peaks, it is important to consider the reactivation and foresee a crisis of the underlying disease at any stage of dengue. Timely diagnosis, an integrated approach, and dynamic management are essential for the successful treatment of both conditions, especially in the general population, particularly in elderly patients.

## Figures and Tables

**Table 1:** Relevant blood test results during hospitalization by date.

Complete blood count	16 Nov	18 Nov	19 Nov	20 Nov	21 Nov	23 Nov	24 Nov	25 Nov	26 Nov	30 Nov
White blood cells (10^3^/mm^3^)	3.7	5.97	6.06	5.7	7.57	6.38	4.55	4.08	3.41	5.75
Red blood cells (10^6^/mm^3^)	1.83	1.65	1.65	1.79	1.89	1.95	2.01	2.54	2.38	2.87
Hemoglobin (g/dL)	6.0	5.4	5.4	5.7	6.4	6.4	6.6	8.19	7.9	9.5
Hematocrit (%)	16.2	15.3	15.4	16.9	18.7	19.6	20.1	25.2	23.4	27.9
MCV (fL)	88.5	92.7	93.3	94.4	98.9	100.5	100	99.2	98.3	97.2
MCH (pg)	32.8	32.7	32.7	31.8	33.9	32.8	32.8	32.3	33.2	33.1
MCHC (g/dL)	37.0	35.3	35.1	33.7	34.2	32.7	32.8	32.5	33.8	34.1
Platelets (10^3^/mm^3^)	101	84	67	58	76	109	124	192	191	296

Abbreviations: CHCM, concentración de hemoglobina corpuscular media; HCM, hemoglobina corpuscular media; VCM, volume corpuscular media.

## Data Availability

The authors have nothing to report.
